# Multi-Target Anti-Psoriatic Effects of *Melissa officinalis*: Phytochemical, Redox, Immunological, and Histological Evidence from an In Vivo Study

**DOI:** 10.3390/molecules31091471

**Published:** 2026-04-29

**Authors:** Branislava Daskalovic, Vladimir Jakovljevic, Sergej Bolevic, Marijana Andjic, Jovana Bradic, Aleksadar Kocovic, Nevena Lazarevic, Vanja Tadic, Ana Zugic, Milos Krivokapic, Nenad Stankovic, Svetlana Trifunovic, Jasmina Sretenovic

**Affiliations:** 1 Department of Physiology, Faculty of Medical Sciences, University of Kragujevac, Svetozara Markovica 69, 34000 Kragujevac, Serbia; 2 Center of Excellence for Redox Balance Research in Cardiovascular and Metabolic Disorders, 34000 Kragujevac, Serbia; 3 Department of Human Pathology, 1st Moscow State Medical University IM Sechenov, Trubetskaya Str. 2, 119992 Moscow, Russia; 4 Department of Pharmacy, Faculty of Medical Sciences, University of Kragujevac, Svetozara Markovica 69, 34000 Kragujevac, Serbia; 5 Institute for Medicinal Plant Research “Dr. Josif Pancic”, Tadeusa Koscuska 1, 11000 Belgrade, Serbia; 6 Faculty of Medicine, University of Montenegro, Kruševac bb, 81000 Podgorica, Montenegro; 7 Independent Researcher, 11000 Belgrade, Serbia; 8 Department of Cytology, Institute for Biological Research “Sinisa Stankovic”—National Institute of Republic of Serbia, University of Belgrade, Bulevar Despota Stefana 142, 11000 Belgrade, Serbia

**Keywords:** psoriasis, *Melissa officinalis*, rat, redox state, morphometry, inflammation

## Abstract

Psoriasis is a chronic inflammatory skin disease linked to immune dysregulation and oxidative stress. Due to the limitations and side effects of conventional therapies, natural compounds are gaining increasing attention. The aim of this study was to evaluate the therapeutic potential of *Melissa officinalis* extract in psoriasis. HPLC analysis was used to characterize the extract, while its antioxidant activity was assessed by DPPH, ABTS, and FRAP assays. An in vivo study was conducted on 18 male *Wistar* albino rats divided into control (CTRL), psoriasis (PSORI), and psoriasis treated with *M. officinalis* (PSORI+MO) groups. Psoriasis was induced by daily topical application of 5% imiquimod cream on the shaved back skin of rats for seven constitutive days. The PSORI+MO group received *M. officinalis* extract orally at a dose of 200 mg/kg during seven days. Redox and inflammatory cytokine analysis were performed. Isolated skin was fixed and stained with H/E and immunohistochemical staining. Morphometric and histological analyses revealed reduced inflammation, keratinocyte proliferation, and epidermal thickness. Systemic and tissue redox status showed decreased oxidative stress biomarkers and enhanced antioxidant defense. Inflammatory cytokines (IL-17A, IL-22, IL-23, IL-1β, and IL-6) were significantly reduced. Our findings suggest that *Melissa officinalis* extract exerts anti-psoriatic effects through antioxidant, anti-inflammatory, and antiproliferative mechanisms.

## 1. Introduction

Psoriasis is a chronic, autoimmune, inflammatory skin disorder characterized by erythema, epidermal thickening, and scaling [1]. It affects approximately 2–3% of the global population and poses a significant public health burden due to its chronic course, frequent relapses, and substantial impact on patients’ quality of life [2,3]. The pathological features of psoriasis include abnormal keratinocyte proliferation, infiltration of diverse immune cell types, and increased vascular hyperplasia [4]. The disease is driven by the interplay of various cells, including neutrophils, macrophages, T lymphocytes, dendritic cells, mast cells, and keratinocytes, which release inflammatory mediators [5]. Alterations in these circulating factors play a central role in disease pathogenesis and are closely associated with the onset and progression of psoriatic lesions [5]. In addition to cutaneous manifestations, psoriasis is often associated with systemic comorbidities such as metabolic syndrome, cardiovascular diseases, and psoriatic arthritis, highlighting its complex pathophysiology [2,3].

In recent years, oxidative stress has been singled out as an increasingly important phenomenon in the pathogenesis of psoriasis. Increased production of reactive oxygen species (ROS) may trigger the release of proinflammatory markers and subsequently damage the keratinocytes and worsen the skin inflammation. Increasing evidence highlights that the imbalance between ROS production and antioxidant defence system mechanisms leads to progression of disease [2,6,7,8]. What is more, the interplay between oxidative stress and cytokine production contributes to maintenance and amplification of chronic inflammation in psoriasis. Excessive ROS production can activate signalling pathways such as NF-κB, thereby inducing increased production of pro-inflammatory cytokines including TNF-α, IL-17, and IL-23. In turn, these cytokines further stimulate immune cells and keratinocytes to produce additional ROS, creating a self-amplifying inflammatory cycle. This reciprocal interaction between oxidative stress and cytokine signaling promotes keratinocyte hyperproliferation, tissue damage, and persistence of psoriatic lesions [6,7,8].

Autoimmune skin disorders, particularly psoriasis, have attracted considerable research interest in recent years, primarily because a fully effective therapeutic strategy for this disease has not yet been established. Although several therapeutic options are currently available, including topical agents, systemic drugs, and biologic therapies, long-term disease management remains challenging due to adverse effects, limited efficacy in some patients, and high treatment costs [9]. Consequently, there is growing interest in complementary therapeutic approaches, particularly plant-derived agents with antioxidant and anti-inflammatory properties, which may provide additional benefits in disease management. Increasing attention has therefore been directed toward herbal-based adjuvant therapies as potential alternatives or complements to synthetic drugs, owing to their favorable safety profile and therapeutic potential. In this context, several herbal preparations have demonstrated beneficial effects in psoriasis in both preclinical and clinical studies [10]. Various types of herbal formulations have been investigated, including topical preparations such as gels, hydrogels, emulsions, and ointments enriched with plant extracts, as well as systemic administration (per os), with the aim of improving pharmacokinetic and therapeutic properties [10].

*Melissa officinalis* L. has been used in traditional medicine for thousands of years due to its numerous health-promoting properties, including sedative, mild hypnotic, antioxidant, anti-inflammatory, cardioprotective, and hypoglycemic effects [11,12]. Since its first description by Pedanius Dioscorides in *De Materia Medica*, the therapeutic use of this plant has persisted, while the range of its potential applications in various pathological conditions has gradually expanded [11]. This plant is a rich source of phenolic acids (such as rosmarinic, caffeic, and coumaric acids), flavonoids, ursolic acid, and triterpenoids [12,13]. Numerous studies have reported the antioxidant, anti-inflammatory, and immunomodulatory properties of *Melissa officinalis*, which are considered largely responsible for its beneficial effects in various diseases [11,12]. Recent findings also suggest that *Melissa officinalis* may exert beneficial effects in the treatment of psoriasis [13,14]. In particular, previous studies investigating both topical [14] and systemic administration [13] of *Melissa officinalis* have demonstrated promising results in the management of this condition.

Considering the abovementioned findings, the present study aimed to evaluate the therapeutic potential of orally administered *Melissa officinalis* extract in an experimental rat model of psoriasis. Particular emphasis was placed on assessing its antioxidant, anti-inflammatory, and antiproliferative effects on psoriatic skin alterations. In addition, the extract was chemically characterized and its in vitro antioxidant activity was evaluated.

## 2. Results

### 2.1. HPLC Characterization of Extract

**Figure 1:**
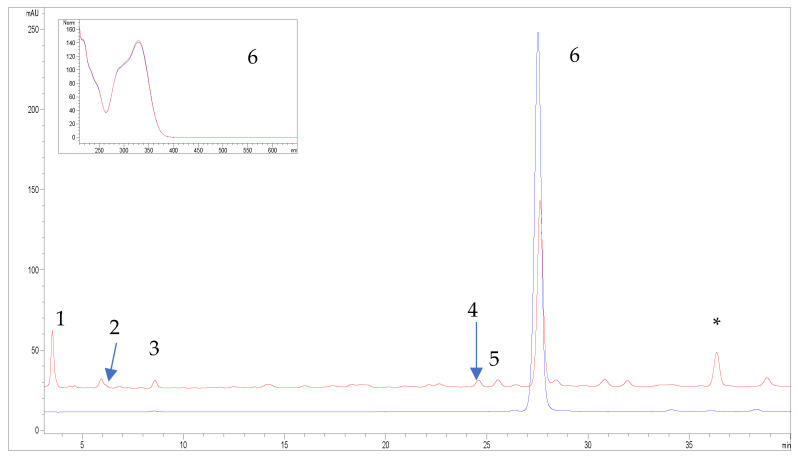
HPLC chromatogram of the *M. officinalis* extract (red line) with the overlaid rosmarinic acid standard (blue line). Peaks are numerically labeled and correspond to the following compounds: (1) protocatechuic acid; (2) neochlorogenic acid; (3) caffeic acid; (4) 3,5-di-O-caffeoylquinic acid; (5) luteolin-7-*O*-glucoside; (6) rosmarinic acid; (*) p-hydroxycinnamic acid derivative (tentative identification). The inset shows the UV/Vis spectra of the rosmarinic acid standard and the corresponding peak (6) from the extract, demonstrating spectral overlap and confirming peak identity.

The results of chemical analysis of the investigated *M. officinalis* extract is present in Table 1 and Figure 1. Six phenolic compounds were identified in the investigated extract. Among them, rosmarinic acid was the dominant compound (462.93 mg/g DW), while the other detected compounds such as neochlorogenic acid, 3,5-di-*O*-caffeoylquinic acid, caffeic acid, luteolin-7-*O*-glucoside and protocatechuic acid were present in considerably lower amounts. The identity of rosmarinic acid (peak 6) was further confirmed by matching both retention time and UV/Vis spectral profile with the reference standard.

### 2.2. Total Tannin, Flavonoid and Polyphenolic Content in M. officinalis Extract

Total tannin, flavonoid and polyphenolic content in *M. officinalis* extract is present in Table 2.

### 2.3. Antioxidant Activity

The antioxidant capacity of LBE was assessed using DPPH and ABTS radical scavenging assays, and the results were compared with those obtained for standard antioxidants, namely ascorbic acid (AA), butylated hydroxyanisole (BHA), and Trolox. The corresponding IC_50_ values are summarized in Table 3.

Significant differences in antioxidant capacity were observed among the tested samples in both assays (one-way ANOVA, *p* < 0.001). The extract exhibited significantly higher IC_50_ values compared with all reference antioxidants in both DPPH and ABTS tests (Tukey post hoc, *p* < 0.001), indicating weaker radical-scavenging activity, while no statistically significant differences were detected among the standard antioxidants (ascorbic acid, BHA, and Trolox)

### 2.4. Effects of the Treatment of Melissa officinalis Extract on the Skin of Psoriatic Rats

**Figure 2:**
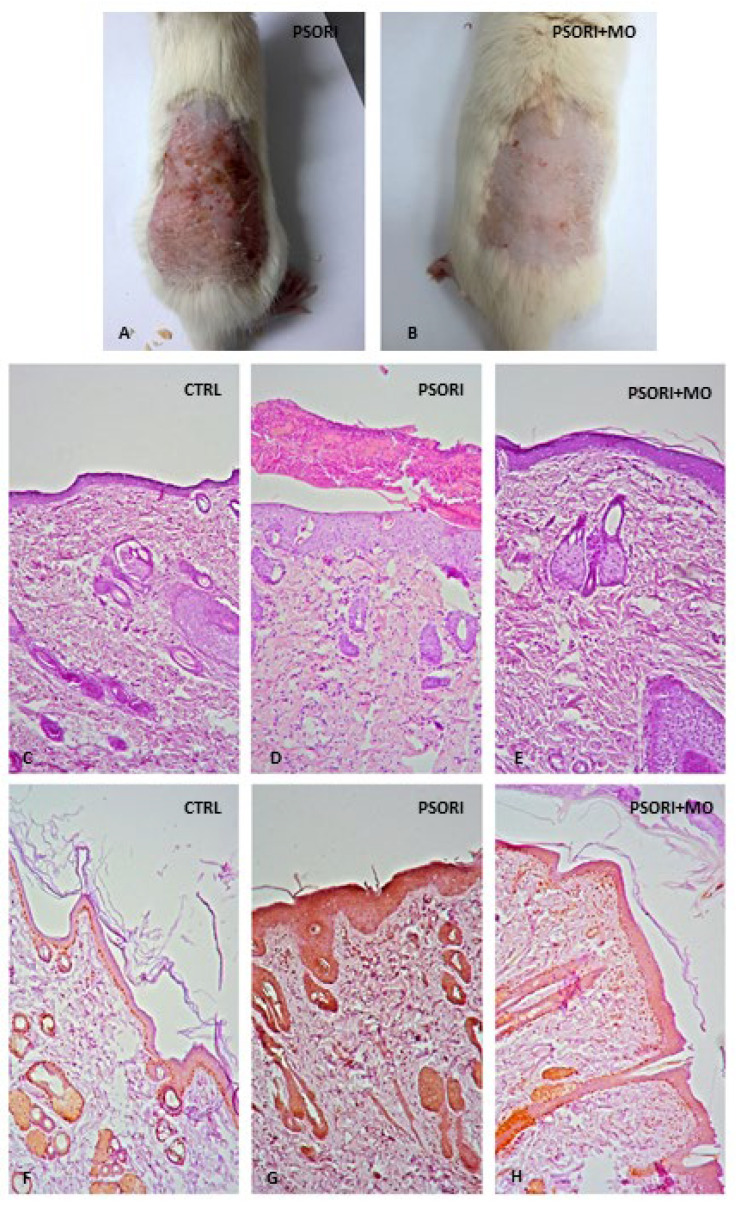
Macroscopic images of psoriatic skin changes in rats (first row): rat skin after psoriasis induction (**A**); rat skin after 7 days of treatment with *M. officinalis* extract (**B**). Microscopic images of rat skin (second and third row) H&E staining: control group skin, (**C**); psoriasis group skin, (D); *Melissa officinalis*-treated group skin, (**E**). PCNA immunohistochemical staining: control group skin, (**F**); psoriasis group skin, (**G**); *Melissa officinalis*-treated group skin (**H**) (Magnification 100×).

Following the induction of psoriasis, the dorsal skin of the rats exhibited pronounced erythema, scaling, and thickening (Figure 2A). After seven days of treatment with *M. officinalis* extract, these clinical manifestations were markedly alleviated, with the skin showing no visible redness or scaling and reduced thickening compared to the PSORI group (Figure 2B). The PASI score after induction was 9, which decreased to 2 following the seven-day treatment period. Histological analysis of skin samples from the control group revealed normal morphology without observable alterations (Figure 2C). In contrast, the PSORI group showed evident desquamation, inflammatory infiltration, and increased epidermal thickness indicative of hyperkeratosis (Figure 2D). In psoriatic rats treated with *Melissa officinalis* extract, histopathological evaluation demonstrated reduced epidermal thickness, diminished inflammation, and decreased desquamation (Figure 2E). In the control group, PCNA immunopositivity was detected in cells of the basal layer of the epidermis, sebaceous glands, and the hair follicle root (Figure 2F). In the PSORI group, immunopositivity was significantly more pronounced, with almost all epidermal cells showing positive staining (Figure 2G). In the PSORI+MO group, PCNA immunopositivity was observed in cells of the basal layer of the epidermis and the hair follicle root (Figure 2H).

### 2.5. Morphometric Analysis

**Figure 3:**
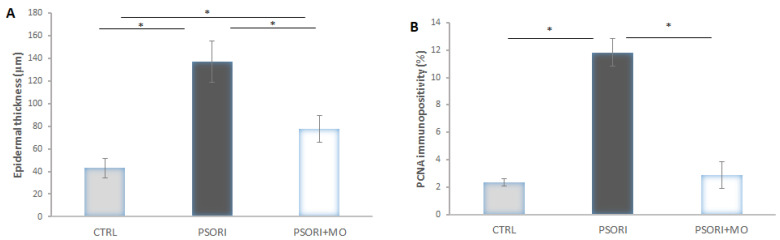
Morphometric analysis of (**A**) epidermal thickness and (**B**) PCNA immunopositivity. Results presented as mean value ± SD (n = 6). Comparison between groups was performed using one-way ANOVA test with the post hoc LSD test analysis (* denotes *p* < 0.05). Control group (CTRL), psoriasis group (PSORI), and psoriasis with *M. officinalis* group (PSORI+MO).

In the PSORI group, an increase in epidermal thickness of 218% was observed, whereas in the PSORI+MO group an increase of 80% was recorded compared to the control values. Treatment with *M. officinalis* reduced epidermal thickening by 76% compared to the PSORI group (Figure 3A). PCNA immunopositivity was increased by 400% in the PSORI group, whereas in the PSORI+MO group it was increased by 22% compared to the CTRL group. In the PSORI+MO group, a decrease in immunopositivity of 308% was observed compared to the PSORI group (Figure 3B).

### 2.6. Tissue Oxidative Stress

**Figure 4:**
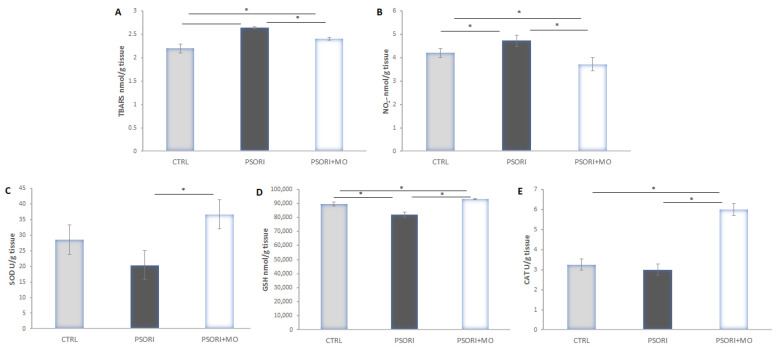
Figure. Oxidative stress in skin. (**A**) TBARS, (**B**) NO_2_^−^, (**C**) SOD, (**D**) GSH and (**E**) CAT. Results presented as mean value ± SD (n = 6). Comparison between groups was performed using one-way ANOVA test with the post hoc LSD test analysis (* denotes *p* ˂ 0.05).

Tissue levels of the pro-oxidative markers TBARS and NO_2_^−^ were significantly increased in the group of animals with psoriasis. Treatment with *Melissa officinalis* extract significantly reduced the levels of these two parameters compared to the untreated psoriasis group. The levels of antioxidant enzymes SOD, GSH, and CAT were decreased in the PSORI group compared to the PSORI+MO group. In contrast to this, administration of the extract significantly increased the levels of antioxidant enzymes compared to the PSORI and CTRL groups (Figure 4).

### 2.7. Systemic Oxidative Stress

**Figure 5:**
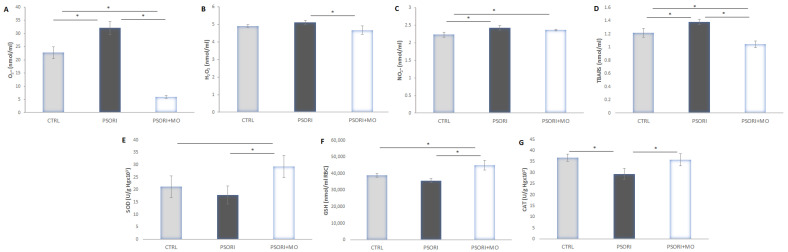
Systemic oxidative stress. (**A**) O_2_^−^, (**B**) H_2_O_2_, (**C**) NO_2_^−^, (**D**) TBARS, (**E**) SOD, (**F**) GSH and (**G**) CAT. Results presented as mean value ± SD (n = 6). Comparison between groups was performed using one-way ANOVA test with the post hoc LSD test analysis (* denotes *p* ˂ 0.05).

An increase in systemic pro-oxidant parameters was observed in the PSORI group, with the most pronounced elevation detected in the levels of superoxide anion radical (O_2_^−^) and the lipid peroxidation index (TBARS) compared to the CTRL group. Following the administration of *Melissa officinalis* extract in psoriatic rats, a significant reduction in O_2_^−^ and TBARS levels was observed compared to the PSORI group. A decrease in catalase activity was also noted in the PSORI group. Conversely, in the PSORI+MO group, a significant increase in systemic levels of SOD, GSH, and CAT was observed compared to the PSORI group (Figure 5).

### 2.8. Inflammatory Cytokines Levels

**Figure 6:**
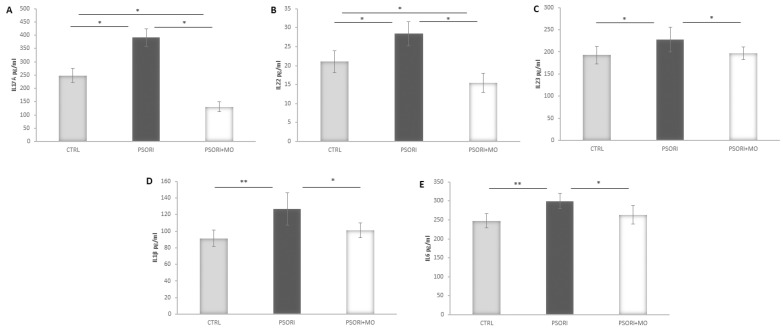
Inflammatory cytokine levels: (**A**) IL17A, (**B**) IL22, (**C**) IL23, (**D**) IL1β and (**E**) IL6. Results presented as mean value ± SD (n = 6). Comparison between groups was performed using one-way ANOVA test with the post hoc LSD test analysis (* denotes *p* < 0.05, ** denotes *p* ˂ 0.005).

The levels of inflammatory cytokines (IL-17A, IL-22, IL-23, IL-1β, and IL-6) were increased in the group of animals with psoriasis. In contrast, treatment with *Melissa officinalis* extract in psoriatic rats significantly reduced the levels of all measured cytokines compared to the control group. The greatest reduction was observed in IL-17A levels, showing a 199% decrease compared to the PSORI group and an 89% decrease compared to the CTRL group. A pronounced reduction was also observed in IL-22 levels, amounting to 84% and 36% compared to the PSORI and CTRL groups, (respectively) (Figure 6).

## 3. Discussion

Plant-derived remedies have been employed for centuries across numerous traditional medical systems, including Traditional Chinese Medicine, European folk practices, and Serbian ethnomedicine, for the management of diverse skin disorders. In the context of psoriasis, a wide range of phytochemicals have shown promising therapeutic effects by modulating central pathophysiological pathways, such as oxidative stress, dysregulated cytokine networks, and excessive keratinocyte proliferation [15]. *M. officinalis* is widely recognized for its use in traditional medicine in the management of various inflammatory conditions [12]. However, data regarding the effects of *M. officinalis* extract in an experimental rat model of psoriasis remain limited. Accordingly, the present study reports the findings of a comprehensive investigation aimed at evaluating the therapeutic potential of *M. officinalis* extract through detailed chemical characterization, assessment of its antioxidant capacity, and in vivo experimental analysis. Within the animal model, particular emphasis was placed on examining the extract’s effects on two critical pathophysiological mechanisms involved in the initiation and progression of psoriasis—on inflammation, proliferation and oxidative stress.

Considering the phytochemical characterization of the *M. officinalis* extract, which revealed a high content of phenolic constituents, predominantly rosmarinic acid along with neochlorogenic acid, 3,5-di-*O*-caffeoylquinic acid, caffeic acid, luteolin-7-*O*-glucoside, and protocatechuic acid, the extract was subsequently subjected to in vitro assays to evaluate its antioxidant capacity. The obtained total phenolic content (TPC) (401.02 ± 0.45 mg GAE/g dry extract) further confirms that the prepared *M. officinalis* extract represents a rich source of phenolic compounds, which are considered the main contributors to its antioxidant potential. In comparison, significantly lower TPC values have been reported in previous studies investigating lemon balm extracts obtained with hydroalcoholic solvents, where phenolic content ranged between approximately 40 and 120 mg GAE/g, depending on extraction conditions and plant origin [16,17,18].

The moderate radical-scavenging activity observed in the DPPH (IC_50_ = 68.21 µg/mL) and ABTS (IC_50_ = 77.94 µg/mL) assays is consistent with literature data, which report IC_50_ values within a comparable range for crude extracts [16,17]. Although significantly weaker than pure reference antioxidants (ascorbic acid, BHA, Trolox), this was expected given the complex phytochemical composition of plant extracts, where antioxidant effects arise from synergistic interactions rather than single highly potent molecules. Importantly, it should be emphasized that direct comparison with pure standards may not fully reflect the biological relevance of plant extracts, as their activity often involves multiple mechanisms beyond direct radical scavenging, including metal chelation, modulation of endogenous antioxidant defenses, and anti-inflammatory effects. Moreover, moderate IC_50_ values do not preclude biological efficacy, particularly in complex biological systems where cumulative and synergistic effects may enhance overall activity.

From a pathophysiological perspective, these findings are particularly relevant considering the established role of oxidative stress in psoriasis, where excessive reactive oxygen species contribute to keratinocyte hyperproliferation and inflammatory signaling [19]. Phenolic constituents of *M. officinalis,* especially rosmarinic acid, have been associated not only with antioxidant but also anti-inflammatory effects [17,18], suggesting a multimodal mechanism of action. Therefore, despite moderate in vitro radical-scavenging activity, the demonstrated phenolic richness and antioxidant capacity provide a rational basis for further incorporation of the extract into a topical cream and its evaluation in an experimental psoriasis model in rats, where combined antioxidant and anti-inflammatory effects may yield more pronounced biological outcomes.

Following comprehensive chemical profiling and previously confirmed antioxidant capacity, the therapeutic potential of orally administered *M. officinalis* extract was further investigated in vivo using a rat model of psoriasis, constituting a logical and necessary step in its preclinical evaluation as a phytotherapeutic agent. The results demonstrated that systemic administration of *M. officinalis* extract markedly attenuated the local cutaneous changes induced by imiquimod in psoriatic rats. Progressive clinical improvement was observed throughout the treatment period, ultimately resulting in a pronounced reduction in erythema, scaling, and epidermal thickening by the end of the study. These clinical observations were substantiated by a significant decrease in the PASI score, confirming the extract’s beneficial effect on the visible manifestations of psoriasis.

Excessive generation of reactive oxygen species (ROS), together with impairment of the antioxidant defense system, plays a crucial role in the initiation and progression of this disorder. Overproduction of ROS can damage key cellular components, including DNA, lipids, proteins, and carbohydrates. Under physiological conditions, a balance is maintained between antioxidant defenses such as SOD, CAT, and GSH and oxidant markers, including MDA, in healthy skin [20]. In our study, we demonstrated that both systemic and skin tissue levels of oxidative stress markers were elevated in the group of rats with psoriasis, which is consistent with previously reported findings [15]. Tissue levels of TBARS and nitrites were increased in animals with psoriasis, further supporting the notion that oxidative stress represents an important pathophysiological mechanism in the development of this disease. A similar trend was observed for systemic oxidative stress parameters, as the levels of measured pro-oxidants (O_2_^−^, H_2_O_2_, TBARS, and nitrites) were significantly increased. On the other hand, treatment with *M. officinalis* significantly reduced both tissue and systemic levels of the measured parameters, suggesting that the beneficial effects of this plant in the treatment of psoriasis may be mediated through the reduction of oxidative stress. Unfortunately, to the best of our knowledge, the available literature does not provide data on the effects of *M. officinalis* on tissue or systemic pro-oxidant levels in the treatment of psoriasis. However, a study by *Draginic* et al. demonstrated that the administration of *M. officinalis* in rats with acute myocarditis reduced systemic levels of O_2_^−^, H_2_O_2_, TBARS, and nitrites [21], which is in agreement with the findings of our study. Regarding antioxidant enzymes, SOD, GSH, and CAT levels were decreased in psoriasis, which is consistent with previous reports [15]. Treatment with *M. officinalis* increased the levels of these enzymes, providing direct evidence of the plant’s antioxidant effects, which were also confirmed through in vitro antioxidant capacity assays. A similar increase in antioxidant enzyme activity following *M. officinalis* treatment has also been reported in a model of acute myocarditis [21].

It is well known that circulating inflammatory cytokines play a central role in the pathophysiology of psoriasis, and changes in the serum levels of inflammatory cytokines are associated with the development of psoriatic lesions and their further progression [5]. This observation was also confirmed in our study, where the levels of the measured cytokines (IL-17A, IL-22, IL-23, IL-1β, and IL-6) were significantly increased in the group of animals with psoriasis. One of the factors contributing to the elevated levels of IL-17A, IL-1β, and IL-6 in psoriasis is increased oxidative stress. Literature data indicate that enhanced oxidative stress leads to the activation of Th1 cells, Th17 cells, and keratinocytes via the MAPK signaling pathway, which results in increased production of these cytokines [4]. In addition, IL-17 promotes inflammation through the induction of T cells and macrophages [5]. These findings were also confirmed in our study, where both tissue and systemic levels of oxidative stress markers were elevated, accompanied by increased inflammation in the skin. The anti-inflammatory potential of *M. officinalis* was demonstrated through the reduction in the levels of inflammatory cytokines. Although all measured cytokines (IL-17A, IL-22, IL-23, IL-1β, and IL-6) showed a significant decrease after treatment with *M. officinalis*, the most pronounced reductions in our study were observed for IL-17A and IL-22. We assume that the decrease in these cytokines may be attributed to rosmarinic acid, which was identified as the most abundant compound in the extract.

Despite the well-documented antioxidant and anti-inflammatory properties of *M. officinalis*, its role in autoimmune diseases remains insufficiently explored in the literature. Evidence suggests that ethanolic extracts of *M. officinalis*, administered orally at a dose of 200 mg/kg, can modulate immune responses, inflammation, and oxidative stress in experimental autoimmune myocarditis in rats [21]. However, in animal models of psoriasis, only a limited number of studies have investigated the therapeutic potential of this plant. One study compared decoction, methanolic, and dichloromethane extracts incorporated into gel formulations and demonstrated anti-psoriatic activity in mice, likely mediated through the antioxidant and anti-inflammatory effects of phytocompounds such as terpenoids and phenolic constituents. In addition, improvements in skin morphology, skin physiology, and barrier function were reported [14]. Furthermore, a clinical study demonstrated anti-psoriatic activity of a herbal syrup administered for 12 weeks. This formulation contains a mixture of Damask rose (*Rosa damascena*), fennel (*Foeniculum vulgare*), lemon balm (*Melissa officinalis*), and honey, prepared according to principles described in Traditional Persian Medicine (TPM). The authors reported significant improvements in itch intensity, Psoriasis Area and Severity Index (PASI), and Dermatology Life Quality Index (DLQI) scores in treated patients; however, the precise molecular mechanisms underlying these effects remain unclear [13].

Additionally, histological analysis in the current study confirmed that psoriasis led to epidermal thickening, hyperkeratosis, keratinocyte proliferation, and the presence of inflammatory infiltration. These findings are consistent with previous studies [15]. In psoriasis, epidermal thickening occurs as a consequence of hyperplasia of basal and suprabasal keratinocytes [22], which can be confirmed by assessing immunoreactivity to PCNA (Proliferating Cell Nuclear Antigen). PCNA is a nuclear protein that is directly associated with cell proliferation, therefore, its quantification is considered an indicator of cellular proliferative activity [23], which was also confirmed in our study. In contrast, treatment with *M. officinalis* reduced epidermal thickening, keratinocyte proliferation, and inflammatory infiltration. The decrease in PCNA immunopositivity further confirmed the antiproliferative effect of this plant. IL-23 is a cytokine that promotes keratinocyte proliferation [5], and in our study, treatment with *M. officinalis* reduced the levels of this cytokine, suggesting that this may represent one of the mechanisms through which the plant exerts its effects. Moreover, the proinflammatory cytokines IL-17A and IL-22 act synergistically to promote keratinization, inflammation, and epidermal thickening [5]. Since treatment with *M. officinalis* reduced epidermal thickness and keratinocyte proliferation, and the most pronounced decrease in proinflammatory cytokine levels was observed for IL-17A and IL-22; these findings directly indicate that the plant exerts its anti-inflammatory effects through this mechanism. This effect was further supported by the observed reduction in inflammatory infiltration. A similar histopathological finding was reported in a study by Dimitris et al., who demonstrated that administration of *M. officinalis* decoction in mice with psoriasis reduced parakeratosis, epidermal thickening, hyperkeratosis, and acute inflammation [14]. Additionally, in psoriasis, cytokines such as IL-17A and IL-22 stimulate fibroblast activation, promoting the recruitment of immune cells and sustaining chronic inflammation [22]. The previous study investigated the effects of *M. officinalis* nanovesicles in inflammatory skin diseases and demonstrated that their therapeutic effects are achieved by restoring cellular energy metabolism and the mitochondrial network, as well as by inhibiting the release of inflammatory mediators [22]. These findings are further supported by our study, which showed a reduction in IL-17A and IL-22 levels.

In addition to rosmarinic acid as the predominant constituent, minor compounds present in our extract, including protocatechuic acid, neochlorogenic acid, caffeic acid, 3,5-di-*O*-caffeoylquinic acid, and luteolin-7-*O*-glucoside, may collectively contribute to the achieved therapeutic effects in psoriasis model. Even though there is no direct evidence for their anti-psoriatic activity, these compounds have been proven to exhibit antioxidant, anti-inflammatory, and keratinocyte-modulating properties in various experimental models [24,25]. Notably, caffeic acid [26] and luteolin derivatives [27] have demonstrated beneficial effects in psoriasis-related inflammatory pathways, further supporting their potential relevance. Thus, the anti-psoriatic efficacy of the extract is likely the result of synergistic interactions among its multiple bioactive constituents rather than the action of rosmarinic acid alone.

## 4. Materials and Methods

### 4.1. Chemicals

All chemicals and analytical-grade reagents used in this study were purchased from Sigma-Aldrich (St. Louis, MO, USA). Absorbance readings were obtained using a BioTek Epoch Microplate Reader (Agilent Technologies, Santa Clara, CA, USA), and the collected data were analyzed with Gen5 software (version 3.17.16).

### 4.2. Plant Material and Preparation of the Extract

Leaves of *Melissa officinalis* L. (Lamiaceae) were utilized for extract preparation. Dried plant material was obtained from Bilje Borča LLC (Belgrade, Serbia), mechanically ground using an IKA A11 mill (Staufen im Breisgau, Germany), and stored in sealed paper bags at ambient temperature prior to extraction. Extraction was performed using 70% ethanol as solvent on the aerial parts of the powdered plant material for 2.5 h. The resulting suspension was filtered through gauze, and the filtrate was allowed to stand at room temperature to facilitate precipitation of insoluble ballast compounds. Afterwards, the supernatant was additionally filtered through Whatman No. 1 filter paper. Solvent removal was carried out under reduced pressure using a rotary evaporator (RV05 Basic IKA, Staufen im Breisgau Germany) at 40 °C, 90 rpm, and 250 mbar until a dry extract was obtained. The final extract was stored in dark glass containers at 4 °C until further analyses [21].

### 4.3. HPLC Analysis

The HPLC fingerprint of the investigated sample was achieved by HPLC (Agilent Technologies 1200). Detection was performed using Diode Array Detector (DAD), and the chromatograms were recorded at λ = 320 nm. HPLC separation of components was achieved using a LiChrospher 100 RP 18e (5 μm), 250 × 4 mm i.d. column, with a flow rate of 0.8 mL/min and mobile phase, A (0.1 M H_3_PO_4_), B (MeCN), elution, combination of gradient mode: 89–75% A, 0–20 min; 75–60% A, 20–35 min; 60–35% A, 35–40 min. A portion of the sample solution (3.01 mg/mL 70% EtOH, DW = 4.1%), previously prepared as described, was filtered through 0.45 μm PTFE filters (Fisher, Pittsburgh, PA, USA) before HPLC analysis. The injected volume was 4 μL. Standard solutions for the determination of polyphenolic compounds were prepared in ethanol at a final concentration of 0.58 mg/mL (protocatechuic acid), 0.34 mg/mL (luteolin-7-*O*-glucoside), 0.39 mg/mL (3,5-di-*O*-caffeoylquinic), 0.52 mg/mL (caffeic acid), 0.3 mg/mL (neochlorogenic acid), and 0.34 mg/mL (rosmarinic acid). A phenolic standard library was constructed by injecting each authentic standard at the aforementioned concentrations, and the data were acquired using a photodiode array detector. The volume injected was 4 μL, the same as the investigated extract. The identification was carried out using retention time and spectral matching. Once spectra matching succeeded, results were confirmed by spiking with respective standards to achieve a complete identification by means of the so-called peak purity test, meaning that each peak was tested for purity by a three-point purity test and for similarity by a library search comparing the peak spectrum to that of the standards. A high similarity index and a common retention time with the standard were considered a positive identification. Those peaks not fulfilling these requirements were not quantified. Under the conditions employed in this study, the relative standard deviation for the retention times in three repetitive runs was in the range of 0.18–1.79%. Quantification was performed by external calibration with corresponding standards. The results represent the mean ± SD of three determinations.

### 4.4. Total Phenolic, Tannin and Flavonoid Content

The total phenolic content was determined by the Folin–Ciocalteu method [28]. One hundred microliters of methanol solution of the investigated sample (with starting concentrations of 5.66 and 6.97 mg/mL, for heaven of tree and poplar, respectively) was mixed with 0.75 mL of 10-fold diluted Folin–Ciocalteu reagent and allowed to stand at 22 °C for 5 min; 0.75 mL of sodium bicarbonate (60 g/L) solution was added to mixture. After 90 min at 22 °C, absorbance was measured at 725 nm. Gallic acid (0–100 mg/L) was used for calibration of a standard curve. The calibration curve showed the linear regression at R2 > 0.99, and the results are expressed as milligrams of gallic acid equivalents per g dry weight (DW). Triplicate measurements were taken and data were presented as mean ± standard deviation (SD).

The percentage content of total tannins (TT) was calculated using the method described in the European Pharmacopoeia 11.0 [29]. Briefly, the investigated extract was treated with phosphomolybdotungstic reagent in an alkaline medium after and without treatment with hide powder reagent. The absorbance was measured by UV-VIS Spectrophotometer HP 8453 (Agilent Technologies, Santa Clara, CA, USA), at a max of 760 nm. The percentage content of tannins expressed as pyrogallol (%, *w*/*w*) was calculated from the difference in absorbance of total polyphenols (A1) and polyphenols not adsorbed by hide reagent (A2), using the following expression:

(62.5 (A1 − A2) × m2)/(A3 × m1)

where m1 represents the mass of the sample to be examined, in grams, and m2 and A2 represent mass, in grams, and the absorbance of pyrogallol, respectively. The results represent the mean of three determinations.

The content of total flavonoids (TF) was calculated using the method described in the European Pharmacopoeia 9.0 [30]. Briefly, the sample was extracted with acetone/HCl under a reflux condenser; the AlCl3 complex of the flavonoid fraction extracted by ethyl acetate was measured by a HP 8453 UV-VIS spectrophotometer, at λmax 425 nm. The content of flavonoid (mean of three determinations), expressed as hyperoside percentage, was calculated using the following expression:

A × 1.25/m

where A was the absorbance at 425 nm and (m) was the mass of the extracts to be examined in grams.

### 4.5. Antioxidative Activity

#### 4.5.1. DPPH Radical Scavenging Assay

The free radical scavenging capacity was evaluated using a modified microplate-based DPPH (2,2-diphenyl-1-picrylhydrazyl) radical scavenging assay adapted for high-throughput analysis. Briefly, a 0.004% (*w*/*v*) solution of DPPH in methanol was combined with appropriate concentrations of the samples or reference antioxidants in 96-well microplates, reaching a total reaction volume of 200 μL in each well. The mixtures were incubated for 30 min at room temperature in the absence of light to allow completion of the reaction. Following incubation, the decrease in absorbance was measured at 515 nm using a microplate reader. The antioxidant potential was expressed as the percentage of DPPH radical inhibition compared to the control containing DPPH solution without any antioxidant compound. Additionally, IC_50_ values, defined as the concentration of sample required to neutralize 50% of DPPH radicals, were calculated for each tested formulation. The validity and reproducibility of the assay were verified through parallel analysis of established antioxidant standards [31].

#### 4.5.2. ABTS Radical Cation Decolorization Assay

Antioxidant activity was further evaluated using the ABTS (2,2′-azino-bis(3-ethylbenzothiazoline-6-sulfonic acid)) radical cation decolorization assay. The ABTS^+^∙ radical was produced by mixing an ABTS stock solution with potassium persulfate and allowing the reaction mixture to stand in the dark for 16 h to ensure complete radical formation. Prior to analysis, the radical solution was diluted with ethanol to obtain an initial absorbance of 0.700 ± 0.020 at 734 nm. For the assay, 20 μL of each tested sample or reference antioxidant was combined with 180 μL of the prepared ABTS^+^∙ working solution in a 96-well microplate. The decrease in absorbance was recorded at 734 nm within one minute after mixing. All determinations were carried out in triplicate to ensure reproducibility and statistical validity of the results [31].

#### 4.5.3. FRAP Assay

The ferric reducing antioxidant power (FRAP) assay was applied to determine the electron-donating capacity of the tested extracts. For each experiment, the FRAP working reagent was freshly prepared by combining 300 mM acetate buffer (pH 3.6), 10 mM TPTZ solution in 40 mM HCl, and 20 mM ferric chloride solution in a volumetric ratio of 10:1:1. In the microplate format, 10 μL of the sample was added to 300 μL of the FRAP reagent in each well. The reaction mixtures were incubated at 37 °C for 10 min to allow the reduction of Fe^3+^–TPTZ complex to its ferrous form. Subsequently, absorbance was measured at 593 nm using a microplate reader. Distilled water was used as a blank control. Trolox served as a positive reference antioxidant, while a calibration curve was generated using ferrous sulfate standards. The antioxidant capacity was expressed as micromoles of Fe^2+^ equivalents (µmol Fe^2+^ eq.) [31].

### 4.6. Experimental Animals

This study was conducted on 18 male Wistar albino rats (10 weeks old, weighing 200–250 g), obtained from the Military Medical Academy in Belgrade, Serbia. The animals were housed individually in transparent plexiglass cages. Environmental conditions were maintained at a constant temperature of 22 ± 1 °C, with a 12 h light/dark cycle. Standard laboratory chow and water were available ad libitum.

The animals were randomly allocated into three groups, each consisting of six rats:

1. CTRL—control group—untreated rats.
2. PSORI—psoriasis.
3. PSORI+MO—psoriasis with systemic administration of *M. officinalis* extract.

### 4.7. Psoriasis Induction and Treatment Protocol

Psoriasis was induced by daily topical application of 50 mg of 5% imiquimod cream (Aldara) to the shaved back skin of rats for seven consecutive days [15]. Animals assigned to the PSORI+MO group received *M. officinalis* extract orally at a dose of 200 mg/kg body weight [22] once daily for seven days, administered by gavage 4 h prior to imiquimod application [15]. At the end of the experimental period, the severity of psoriatic lesions was assessed using the Psoriasis Area and Severity Index (PASI). Erythema, scaling, and skin thickness were evaluated and scored independently on a scale ranging from 0 to 4 (0 = none; 1 = mild; 2 = moderate; 3 = marked; 4 = very marked), yielding a maximum cumulative PASI score of 12 [15].

### 4.8. Histology, Immunohistochemistry and Morphometry

Skin specimens were fixed in 4% neutral-buffered formalin and subsequently dehydrated using a graded ethanol series (70%, 96%, and 100%). After clearing in xylene, the samples were embedded in Histowax^®^ (Histolab Product AB, Göteborg, Sweden) for histological analysis. Sections 5 µm thick were prepared and stained with hematoxylin and eosin (H&E) to evaluate morphological changes [15]. PCNA expression was evaluated using an immunohistochemical staining method. After deparaffinization and rehydration, the sections were rinsed in 0.01 M phosphate-buffered saline (PBS; pH 7.6) for 10 min. Antigen retrieval was performed by heating the sections in a microwave oven (700 W) in 0.05 M citrate buffer (pH 6.0) for two cycles of 10 min each. To inhibit endogenous peroxidase activity, the sections were incubated for 15 min in a hydrogen peroxide solution prepared in methanol and subsequently washed in PBS for 10 min. The samples were then preincubated with normal goat serum (1:10) for 30 min to prevent nonspecific binding, followed by overnight incubation with the primary anti-PCNA antibody (1:500; 60097-1-Ig, Proteintech, Proteintech Group, Wuhan, China). After rinsing in PBS, the sections were incubated for 1 h with the secondary antibody (polyclonal goat anti-mouse; 1:200; ab6789, Abcam, Cambridge, UK) and washed again in PBS. Immunoreactivity was visualized using a 0.05% 3,3-diaminobenzidine tetrachloride (DAB) liquid substrate chromogen system [32]. Digital micrographs of the tissue sections were obtained using a digital camera attached to an Olympus BX51 microscope (Olympus Corporation, Tokyo, Japan). Morphometric evaluation was conducted using Image Pro-Plus software 7.0. (Media Cybernetics, Rockville, MD, USA) [15,33].

### 4.9. Skin Homogenization

Skin samples obtained from all experimental animals were preserved at −80 °C until further processing. Prior to analysis, tissues were homogenized on ice in phosphate-buffered saline (PBS, pH 7.4) at a ratio of 1:10 (*w*/*v*) using an electric homogenizer. The resulting homogenates were centrifuged at 1200× *g* for 20 min at 4 °C, after which the supernatants were carefully collected [15].

### 4.10. Oxidative Stress Analysis

In the collected plasma samples, the levels of lipid peroxidation (TBARS), nitrite concentration (NO_2_^−^), superoxide anion radical (O_2_^−^), and hydrogen peroxide (H_2_O_2_) were determined, whereas in erythrocyte lysate samples the activities of superoxide dismutase (SOD), catalase (CAT), and reduced glutathione (GSH) were measured according to our previously described methodology (dodati neciju referencu). In skin homogenate samples, the levels of lipid peroxidation (TBARS), nitrite concentration (NO_2_^−^), and the activities of superoxide dismutase (SOD), catalase (CAT), and reduced glutathione (GSH) were also determined according to our previous research protocol [15]. All samples were subsequently analyzed using a Shimadzu UV-1800 spectrophotometer (Kjota, Japan).

### 4.11. Inflammatory Cytokine Analysis

Concentrations of IL-1β, IL-6, IL-22, IL-23, and IL-17A were quantified in collected serum samples. Serum levels of IL-1β, IL-6, IL-22, and IL-23 were measured using rat ELISA kits (FineTest, Wuhan Fine Biotech Co., Ltd., Wuhan, China, Cat. No: ER 1094; Cat No: ER 0042; Cat No: ER 1630; Cat No: ER 1096, respectively), whereas IL-17A levels were determined using a rat ELISA kit from Reed Biotech (Wuhan, China, Cat. No: RE2867R). The assays were performed on microtiter plates pre-coated with monoclonal antibodies specific to each cytokine. Standards with known concentrations and serum samples were added to the wells and incubated with the corresponding biotin-labeled detection antibodies. Color development was proportional to the cytokine concentration present in the samples. Absorbance was measured at 450 nm using an ELISA plate reader (UT-2100C, MCR lab, Holon, Israel). Cytokine concentrations were expressed in pg/mL. The assay detection ranges were 31.25–2000 pg/mL for IL-1β, 62.5–4000 pg/mL for IL-6, 15.625–1000 pg/mL for IL-22, 15.625–1000 pg/mL for IL-23, and 15.63–1000 pg/mL for IL-17A.

### 4.12. Statistical Analysis

The normality of data distribution was assessed using the Shapiro–Wilk test. Statistical analyses were carried out using SPSS software (version 20.0; SPSS Inc., Chicago, IL, USA). Differences among groups were evaluated by one-way ANOVA followed by the LSD and Tukey post hoc test for multiple comparisons. A *p*-value of less than 0.05 was considered statistically significant. Results are expressed as mean ± standard deviation (SD).

## 5. Conclusions

Overall, the present investigation demonstrates that *M. officinalis* extract possesses considerable therapeutic potential in the treatment of psoriasis, as evidenced by its antioxidant, anti-inflammatory, and antiproliferative properties. First, the results revealed a marked improvement in visible macroscopic psoriatic skin lesions, reflected by a reduction in erythema, epidermal thickening, and scaling. Moreover, significant improvement in the histopathological features of psoriasis was observed, including decreased epidermal thickness, reduced keratinocyte proliferation, and diminished inflammation. Second, our findings suggest that some of the key mechanisms underlying the beneficial effects of *M. officinalis* in psoriasis involve the modulation of oxidative stress and inflammatory processes. The total therapeutic efficacy of the extract in this study may be explained by synergistic activity of its major constituent, rosmarinic acid, together with other less abundant phenolic compounds, including protocatechuic acid, neochlorogenic acid, caffeic acid, 3,5-di-*O*-caffeoylquinic acid, and luteolin-7-*O*-glucoside. Nevertheless, further studies are required to elucidate additional mechanisms, beyond those identified in the present study, through which *M. officinalis* may exert its therapeutic effects in psoriasis.

## Figures and Tables

**Table 1:** Chemical profile of the investigated *M. officinalis* extract obtained by applying HPLC analysis.

Peak Number	Identified Compounds	Content mg/g DW of the Investigated Extract
1	Protocatechuic acid	3.60
2	Neochlorogenic acid	15.93
3	Caffeic acid	7.91
4	3,5-di-*O*-caffeoylquinic acid	15.36
5	Luteolin-7-*O*-glucoside	4.18
6	Rosmarinic acid	462.93

**Table 2:** Determination of major polyphenolic compounds of *M. officinalis* extract.

*M. officinalis* Extract	
Tannin content (%)	8.25 ± 0.10
Total phenolic content (mg GAE/g DW of extract)	401.02 ± 0.45
Total flavonoid (%)	0.43 ± 0.04

**Table 3:** IC_50_ values of antioxidant activity of the tested samples determined by DPPH and ABTS assays.

Investigated Samples and Standards	DPPH IC_50_ (µg/mL)	ABTS IC_50_ (µg/mL)
MOE	68.21 ± 5.96 ^a^	77.94 ± 8.12 ^a^
AA	8.27 ± 0.71 ^b^	9.55 ± 0.82 ^b^
BHA	10.71 ± 0.95 ^b^	12.57 ± 1.04 ^b^
Trolox	4.78 ± 0.15 ^b^	6.18 ± 0.67 ^b^

Values are presented as mean ± SD (n = 3). Different superscript letters (a, b) within the same column indicate statistically significant differences according to one-way ANOVA followed by Tukey’s post hoc test (*p* < 0.001). Lower IC_50_ values indicate stronger antioxidant activity. DPPH—2,2-diphenyl-1-picrylhydrazyl radical scavenging assay; ABTS—2,2′-azino-bis(3-ethylbenzothiazoline-6-sulfonic acid) radical cation decolorization assay; AA—ascorbic acid; BHA—butylated hydroxyanisole; Trolox—water-soluble vitamin E analogue (6-hydroxy-2,5,7,8-tetramethylchroman-2-carboxylic acid).

## Data Availability

Data are contained within the article.
